# Internet‐based cognitive behaviour therapy for the prevention, treatment and relapse prevention of eating disorders: A systematic review and meta‐analysis

**DOI:** 10.1002/pchj.715

**Published:** 2023-12-17

**Authors:** Nilima Hamid

**Affiliations:** ^1^ Centre for Medical Education Cardiff University School of Medicine Cardiff UK

**Keywords:** anorexia nervosa, binge‐eating disorder, bulimia nervosa, cognitive behaviour therapy, eating disorder

## Abstract

Eating disorders (EDs) are undertreated worldwide. In the UK the lag between recognition of symptoms and treatment ranges from about 15 months to in excess of 2 years. Internet‐based cognitive behaviour therapy (ICBT) could be a viable alternative to face‐to‐face cognitive behaviour therapy (CBT) that avoids the negative impacts of delayed interventions. Based on evidence from randomised controlled trials (RCTs), this systematic review investigated the efficacy of minimally guided self‐help ICBT, without face‐to‐face therapy, for the prevention, treatment and relapse prevention of all types of EDs in adults. The electronic databases MEDLINE, PsychINFO, CENTRAL, Scopus, and Web of Science were searched between 1991 and 2021. Inclusion criteria specified RCTs with ICBT versus inactive comparison groups. The Cochrane Risk of Bias Tool‐2 was used for quality assessments. Qualitative synthesis and meta‐analyses were conducted. Findings typically showed medium significant beneficial effect sizes for prevention studies ranging from (−0.31 [95% CI: −0.57, −0.06] to −0.47 [95% CI: −0.82, −0.11]) and generally large effect sizes for the treatment studies ranging from (−0.30 [95% CI: −0.57, −0.03] to −1.11 [95% CI: −1.47, −0.75]). Relapse prevention studies yielded mainly small non‐significant beneficial effects with significant effect sizes of (−0.29 [95% CI: −0.56, −0.03] and −0.43 [95% CI: −0.70, −0.16]). Only the treatment studies reached clinical significance and cognitive symptoms improved more than behavioural symptoms. ICBT appears to be efficacious for the prevention, treatment and relapse prevention of eating disorders with treatment interventions being the most beneficial. However, the evidence base is very small, particularly for treatment and relapse prevention, indicating the need for more high‐quality RCTs.

## INTRODUCTION

Eating disorders (EDs) are undertreated worldwide (Fairburn & Patel, 2014). In the UK the lag between recognition of symptoms and treatment ranges from about 15 months to in excess of 2 years; this is a concern as there is evidence that early treatment leads to better outcomes (Beat, 2015). Face‐to‐face cognitive behaviour therapy (CBT) is the primary treatment for EDs in adults but its availability is limited with long waiting lists due to lack of trained therapists and costs can be high (Watson et al., 2018). Internet‐based cognitive behaviour therapy (ICBT) could provide a cost‐effective evidence‐based alternative to face‐to‐face CBT that avoids the negative impacts of delayed interventions.

The negative consequences of untreated EDs include many physical and mental health complications such as diabetes, hypertension, depression and anxiety. Individuals with an ED have higher standardised mortality ratios (SMRs), increased suicide rates, poorer quality of life than the general population, and EDs are linked to increased healthcare burden (Ágh et al., 2016). Internet‐based CBT could broaden the reach of evidence‐based interventions for EDs.

Although it is difficult to deal immediately with crises such as suicidal ideation or other adverse events with ICBT, such programmes are appealing due to being cheaper, briefer, more accessible regarding time constraints and geographical barriers, and they are more convenient than face‐to‐face CBT. In addition, those refraining from seeking treatment for fear of stigma associated with EDs may find the anonymity offered by ICBT more acceptable than face‐to‐face treatment and thus may seek help sooner (Ali et al., 2017).

Watson et al. (2018) reported that the cost‐effectiveness of ICBT was comparable to face‐to‐face CBT for EDs. Thus, even if small effect sizes are found for ICBT, minimally guided ICBT could be potentially highly scalable and this would free resources to treat patients who need more intensive treatment. The importance of ICBT was highlighted by the COVID‐19 pandemic, which increased the risk of developing an ED and exacerbated existing EDs in at‐risk groups globally, thus leading to a demand for remote interventions such as ICBT during a period of limited access to face‐to‐face healthcare services (Rodgers et al., 2020).

Based on evidence from randomised controlled trials (RCTs), this systematic review investigated the efficacy of minimally guided self‐help ICBT, without face‐to‐face therapy, for the prevention, treatment and relapse prevention of all types of EDs in adults.

## METHODS

This systematic review was conducted as part of the dissertation submission for the award of MSc in Psychiatry (Cardiff University, UK) and in accordance with the preferred reporting items for systematic reviews and meta‐analyses (PRISMA) guidelines (Moher et al., 2009). The search, screening, quality assessment and data extraction processes were undertaken independently under supervision.

### Eligibility criteria

Using the PICOS framework (Moher et al., 2009) inclusion criteria were: *Population*: Adults with an ED. *Intervention*: ICBT—interactive, multimedia, online structured CBT programmes. *Comparator*: Inactive comparison groups. *Outcome*: Prevention, treatment and relapse prevention of EDs. *Study Design*: RCTs published in peer‐reviewed journals—not study protocols, nor grey literature.

Only inactive comparisons (i.e., waiting‐list, delayed‐treatment and treatment‐as‐usual controls) were included to reduce heterogeneity as scoping searches showed active comparisons to be diverse and sparse. There were no geographical or gender restrictions, but non‐English language reports were excluded.

### Terminology: Intervention types

Interventions for eating disorders are on a spectrum based on the risk of target population: (prevention) universal, selective, indicated; (treatment) case identification, standard treatment for known disorders; (maintenance) compliance with long‐term treatment and aftercare (Haggerty & Mrazek, 1994).

In this systematic review prevention programmes are those that occur before the first onset of an ED and include universal prevention targeted at whole populations at risk; selective prevention focuses on high‐risk populations; and indicated prevention is directed at sub‐threshold EDs. Treatment programmes occur after the onset of an ED and relapse prevention programmes are implemented post‐treatment to maintain beneficial outcomes.

### Search strategy

The electronic databases MEDLINE, PsychINFO, CENTRAL, Scopus and Web of Science were searched between 1991 and 2017 on 26 October 2017, with an updated search on 27 January 2021. Although phrase searching was not used, the search was thorough and the keywords searched in the titles and abstracts for each database are shown in Table 1. Citations and references in relevant reports were used to identify further reports.

**TABLE 1:** Search strategy for databases.

Keywords
Eating disorders or “feeding and eating disorders” or anorexia nervosa or anorexia or bulimia nervosa or bulimia or binge eating disorder or EDNOS Cognitive therapy or CBT or cognitive behavio* therap* or e therap* or online or electronic or internet or web based 1 and 2

*Note*: Eating Disorder Not Otherwise Specified (EDNOS) is an ED that does not meet the full criteria for Anorexia Nervosa (AN), Bulimia Nervosa (BN) or Binge‐Eating Disorder (BED), and is defined in the Diagnostic and Statistical Manual of Mental Disorders, DSM‐5 (American Psychiatric Association, 2013) as ‘Other Specified Feeding or Eating Disorder’ (OSFED) and ‘Unspecified Feeding or Eating Disorder’ (UFED).

### Quality assessments

The risk of bias in the included studies was critically appraised using the Cochrane Risk of Bias Tool (Higgins & Green, 2008) and updated using RoB 2 (Sterne et al., 2019). The latter rates studies as at *low risk of bias*, *some concerns* or *high risk of bias* in five domains along with an overall risk of bias judgement:

1. Risk of bias arising from the randomisation process.
2. Risk of bias due to deviations from the intended interventions *(effect of assignment or adherence to intervention)*.
3. Risk of bias due to missing outcome data.
4. Risk of bias in measurement of the outcome.
5. Risk of bias in the selection of the reported result.

### Data extraction and analysis

Study and participants' characteristics, as well as means and standard deviations for selected outcomes from the included studies were extracted into Microsoft Word tables. Following qualitative synthesis, random effects meta‐analyses were conducted using Review Manager 5.3 (Cochrane Collaboration, 2014). Standardised mean difference (SMD), in this case Hedges' (adjusted) *g* (Higgins & Green, 2008), was the (post‐treatment between group) effect size used in the meta‐analyses and was interpreted according to the thresholds provided by Cohen (1988) of 0.2 = small effect size, 0.5 = medium effect size, and 0.8 = large effect size. When there were insufficient data for a meta‐analysis, individual effect sizes were calculated if data permitted.

The extent of heterogeneity, *I* ^2^ (0%–100%) was calculated. *I* ^2^ thresholds are low at <40%; moderate at 30%–60%; substantial at 50%–90%; and considerable at 75%–100% (Higgins & Green, 2008).

In addition, the strength of the whole body of evidence was assessed using the grades of recommendation, assessment, development and evaluation (GRADE) approach. Confidence in the effect estimates can be categorised as ‘high’, ‘moderate’, ‘low’ or ‘very low’. RCTs are initially classed as ‘high’ level of evidence but can be reduced by a maximum of three levels based on five criteria (indirectness; study limitations—risk of bias; inconsistency; imprecision; and publication bias) (Higgins & Green, 2008).

Various standardised, reliable and validated instruments which are established in the EDs field were consistently used in the included studies. The outcome measures selected for meta‐analysis are shown in Table 2.

**TABLE 2:** Outcome measures (in included studies) selected for meta‐analysis (higher scores indicate worse ED psychopathology).

Outcome measure	Description
Eating disorder examination questionnaire (EDE‐Q) (Fairburn & Beglin, 1994)	Measures eating disorder cognitive symptoms (restraint and eating, weight, shape concerns, global score), and behavioural items (binge and purge rates) in the past 28 days.
Eating disorder inventory (EDI/EDI‐2) (Garner, 1991; Garner & Olmsted, 1984)	Assesses the subscales: drive for thinness (fear of gaining weight); bulimia (binge‐eating and purging behaviour); body dissatisfaction; ineffectiveness (not feeling control over life‐low self‐esteem); perfectionism; interpersonal distrust; interoceptive awareness (sensations of hunger and satiety); maturity fears; impulse regulation; social insecurity; asceticism; and total score.
Weight concern scale (WCS) (Killen et al., 1994)	Determines weight concerns linked to body image.
Body shape questionnaire (BSQ) (Cooper et al., 1987)	Rates body shape concerns.
Body attitude test (BAT) (Probst et al., 1995)	Measures ‘negative appreciation of body size; lack of familiarity with one's own body; general body dissatisfaction; and a rest factor’.
Beck depression inventory (BDI‐2) (Beck et al., 1988)	Assesses severity of depression.
Centre for Epidemiological Studies Depression scale (CES‐D) (Orme et al., 1986)	Determines severity of depression.
Symptom checklist 90R (general symptom index score) (SCL 90R GSI) (Derogatis, 1975)	Rates general psychopathology/anxiety.

None of the meta‐analyses included a minimum of 10 studies, thus no funnel plots were undertaken (Higgins & Green, 2008, p. 317). Instead, publication bias was explored by comparing fixed and random effects estimates and visually inspecting forest plots for trends indicating possible publication bias (Higgins & Green, 2008, p. 321). Sensitivity analyses were performed to evaluate the robustness of the results to variations in quality of the studies, study size, type of ICBT programme, level of risk of ED, type of ED, and to explore heterogeneity.

## RESULTS

**FIGURE 1:**
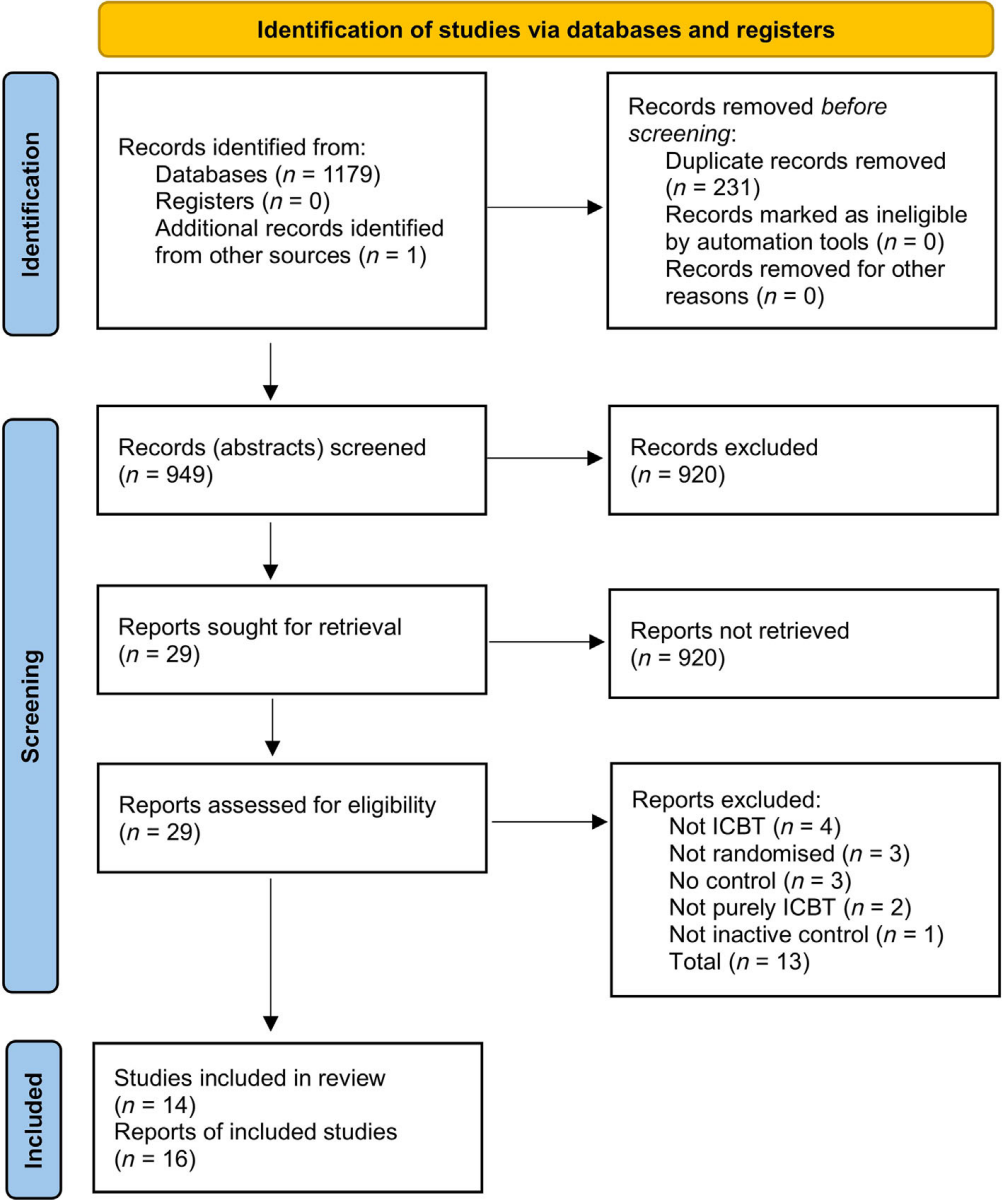
PRISMA flow diagram (Page et al., 2021). ICBT, internet‐based cognitive behaviour therapy; PRISMA, Preferred reporting items for systematic reviews and meta‐analyses.

In all 1180 reports were identified, 949 abstracts were screened and 29 reports were assessed for eligibility. Fourteen RCT studies presented in 16 reports were included in this systematic review (*N* = 2076). Figure 1 describes the study selection process.

### Cochrane Risk of Bias Tool (RoB 2)

Both the initial assessment and RoB 2 (Sterne et al., 2019) identified the same studies as at low risk of bias (Jacobi et al., 2017; Sánchez‐Ortiz et al., 2011; Taylor et al., 2016); however, Fichter et al. (2012) was identified as at low risk of bias by RoB 2 but not by the initial assessment. The *risk of bias due to deviations from the intended interventions* (*domain 2*) was considered low for all the treatment and relapse prevention studies, whereas for the prevention studies risk of bias for this domain varied across studies. Although the evidence base is small for the treatment and relapse prevention studies, they seem to be more consistently of better quality than the prevention studies (Table 3).

**TABLE 3:** Cochrane risk of bias tool (ROB2): assessment of risk of bias in included studies (Sterne et al., 2019).

Study	Domain 1: Risk of bias arising from the randomisation process	Domain 2: Risk of bias due to deviations from the intended interventions (effect of assignment to intervention)	Domain 3: Risk of bias due to missing outcome data	Domain 4: Risk of bias in measurement of the outcome	Domain 5: Risk of bias in selection of the reported result	Overall risk of bias
**Prevention studies**						
Jacobi et al. (2007)						
Jacobi et al. (2012) and Völker et al. (2014)						
Low et al. (2006)						
Saekow et al. (2015)						
Taylor et al. (2006)						
Taylor et al. (2016)						
Winzelberg et al. (2000)						
Zabinski et al. (2001)						
**Treatment studies**						
Carrard et al. (2011)						
Ruwaard et al. (2013)						
Sánchez‐Ortiz et al. (2011)						
Wagner et al. (2016)						
**Relapse prevention studies**						
Fichter et al. (Fichter et al., 2012, 2013)						
Jacobi et al. (2017)						

*Note*: Green, low risk of bias; Red, high risk of bias; Yellow, some concerns.

When interpreting the outcome of quality assessments, such as RoB 2 (Sterne et al., 2019), it is important to consider whether poor reporting was likely to influence the scores. Moreover, it is necessary to note that blinding of participants and personnel is practically impossible in psychotherapy trials, particularly versus no treatment.

None of the RCTs included in this review were double‐blinded and only six studies used blinded outcome assessment (Fichter et al., 2012; Jacobi et al., 2012, 2017; Sánchez‐Ortiz et al., 2011; Taylor et al., 2006, 2016). Blinded outcome assessment is particularly important when participants and care providers cannot be blinded, as with psychotherapy trials versus no treatment, and for outcomes which are subjective as opposed to objective. Blinded outcome assessment thwarts detection bias but increases cost and complexity of the trial. Blinding terminology used in reporting tends to be ambiguous (Schulz & Grimes, 2002). For example, *double‐blinding*, unless clearly stated, could refer to any two of three groups: participants, healthcare provider or outcome assessor. Blinded outcome assessment is poorly reported and often not used despite being possible. The success of blinding is rarely assessed (Kahan et al., 2015). Reports of blinding methods used in trials should be explicit and in accordance with the consolidated standards of reporting trials (CONSORT) statement (Schulz et al., 2010). According to Hrobjartsson et al. (2014), non‐blinded patient reported outcome assessment may be even more biased than those by non‐blinded outcome assessors for subjective outcomes. Thus, the possibility of biased overestimates is of concern in this review, as the included studies mostly assessed subjective outcomes via non‐blinded patient self‐report measures.

### Study and participants' characteristics

The ICBT ED prevention, treatment and relapse prevention programmes are interactive, multimedia, online interventions based on CBT principles with modules consisting of psychoeducation, cognitive restructuring, behavioural modification and relapse prevention, all reinforced by homework assignments and email support. Prevention studies also included an asynchronous discussion group and relapse prevention studies offered real‐time online chat sessions (Table 4).

**TABLE 4:** Study and participants' characteristics.

Study	Setting/sample type	Intervention	Control	Duration	Follow‐up period	Frequency of therapist contact
**Prevention studies**						
Jacobi et al. (2007)	Two German universities' students/self‐referred (via adverts, university email)	*Student Bodies* (SB) adapted for a German population	WLC	8 weeks	3 months	Weekly email; moderated asynchronous anonymous (AA) discussion group
Völker et al. (2014)	Four German universities' students/self‐referred (via adverts, university email)	*Student Bodies* adapted for women with sub‐threshold ED (SB+)	WLC	8 weeks	6 months	Weekly email; moderated AA discussion group
Low et al. (2006)	US (North‐East) undergraduate college students/self‐referred (via email adverts)	*Student Bodies* with/without moderated discussion group/no discussion group	WLC	8 weeks	8–9 months	Weekly email; moderated AA discussion group. *Or* weekly email support with un‐moderated/no discussion group
Saekow et al. (2015)	US private university students; from community/self‐referred (via adverts; university email)	*Student Bodies* adapted to target restrictive eating, bulimic and compensatory behaviour (SB‐ED)	DTC	10 weeks	None	Weekly email; moderated AA discussion group
Taylor et al. (2006)	US college students California (San Diego; San Francisco Bay)/self‐referred (via adverts, campus mailings, mass media)	*Student Bodies*	DTC	8 weeks	3 years	Weekly email; moderated AA discussion group
Taylor et al. (2016)	Fourteen US colleges‐universities' students/self‐referred (via adverts; emails)	Image and Mood (IaM) adapted from *Student Bodies*	DTC	10 weeks	2 years	Weekly email; moderated AA discussion group; two individualised feedback emails over 10 weeks
Winzelberg et al. (2000)	US (West Coast) public university students/self‐referred (recruited via adverts)	*Student Bodies*	DTC	8 weeks	3 months	Weekly email; moderated AA discussion group
Zabinski et al. (2001)	US (West Coast) public university students/self‐referred (from university psychology course)	*Student Bodies*	DTC	8 weeks	10 weeks	Weekly email; moderated AA discussion group
**Treatment studies**						
Carrard et al. (2011)	Switzerland (Geneva). Community/self‐referred (via adverts in magazines, newspapers; two health websites)	ICBT programme SALUT‐BED adapted from the SALUT‐BN programme	DTC	6 months	6 months	Weekly email; three face‐to‐face meetings for assessments. Individualised feedback according to need
Ruwaard et al. (2013)	Dutch community/self‐referred (via adverts)	ICBT programme for BN (Interapy) or Bibliotherapy (unguided)	DTC	20 weeks	1 year	‘Intensive’ (13 h total per participant) email support individualised according to need
Sánchez‐Ortiz et al. (2011)	Six UK (London) higher education institutions' students/self‐referred (via adverts; emails)	ICBT programme Overcoming Bulimia Online (OBO)	DTC	3 months	3 months	Weekly email support. Individualised feedback according to need
Wagner et al. (2016)	German, Austrian, Swiss community/self‐referred (via adverts and clinical sample)	ICBT programme for BED (ICBT‐BED)	DTC	16 weeks	3, 6, 12 months	‘Intensive’ (11 h total per participant) email feedback to writing assignments
**Relapse prevention studies**						
Fichter et al. (2012; 2013)	AN patients discharged from inpatient treatment/clinical sample from eight German hospitals	ICBT programme (VIA) for relapse prevention of AN	TAU	9 months	9 months	Weekly email individualised to needs (tapered off), moderated discussion group, monthly online (synchronous) 1 h group chat
Jacobi et al. (2017)	BN patients discharged from inpatient treatment/clinical sample from 13 German hospitals	ICBT programme (IN@) for relapse prevention of BN	TAU	9 months	9 months	Weekly email individualised to needs; monthly online (synchronous) 1 h individual chat

Study	Participants (Intervention vs. Control)	Mean age‐years (SD) (Range)	Ethnicity	Socioeconomic status	Diagnosis (DSM‐4)
**Prevention studies**					
Jacobi et al. (2007)	*N* = 100	Intervention 22.5 (2.7) (NR)	NR (German university student population)	NR	None
	(50/50)	Control 22.1 (2.6) (NR)			
	All female	Total sample NR (NR) (NR)			
Jacobi et al. (2012); Völker et al. (2014)	*N* = 126	Intervention NR (NR) (NR)	NR (German university student population)	NR	None
	(64/62)	Control NR (NR) (NR)			
	All female	Total sample 22.3 (2.9) (18–33)			
Low et al. (2006)	*N* = 72	Intervention NR (NR) (NR)	Non‐Caucasian 8.4%	NR	None
	(NR)	Control NR (NR) (NR)			
	All female	Total sample NR (NR) (NR)			
Saekow et al. (2015)	*N* = 65	Intervention NR (NR) (NR)	Caucasian 56.1%, Chinese 19.5%, African American 9.8%, Japanese 9.8%, Latino/Hispanic 7.3%, Asian Indian 4.9%, Mexican American 4.9%, Korean 2.4%, Pacific Islander 2.4%, Vietnamese 2.4%, Other 12.2%	NR	Subclinical
	(31/34)	Control NR (NR) (NR)			ED
	All female	Total sample NR (NR) (18–25)			(DSM‐5)
Taylor et al. (2006)	*N* = 480	Intervention NR (NR) (NR)	Caucasian 60%, African American 2%, Hispanic 10%, Asian 17%, Other 11%	NR	None
	(244/236)	Control NR (NR) (NR)			
	All female	Total sample 20.8 (2.6) (17–31)			
Taylor et al. (2016)	*N* = 206	Intervention 20.2 (1.8) (NR)	Caucasian 51%, African American 11%, Hispanic 10%, Asian American 21%, Other 7%	NR	None
	(106/100)	Control 20.5 (1.9) (NR)			
	All female	Total sample NR (NR) (NR)			
Winzelberg et al. (2000)	*N* = 60	Intervention NR (NR) (NR)	Caucasian 53%, Hispanic 35%, Asian 5%, African American 3%, Other 3%	NR	None
	(31/29)	Control NR (NR) (NR)			
	All female	Total sample 20.0 (2.8) (18–33)			
Zabinski et al. (2001)	*N* = 62	Intervention NR (NR) (NR)	Caucasian 66.1%, Latina/Hispanic 26.8%, Asian 1.8%, Other 5.4%	NR	None
	(31/31)	Control NR (NR) (NR)			
	All female	Total sample 19.3 (1.4) (17–24)			
**Treatment studies**					
Carrard et al. (2011)	*N* = 74	Intervention 34.4 (11.0) (NR)	NR (Swiss community population)	NR	BED full syndrome/ sub‐threshold
	(37/37)	Control 37.8 (11.8) (NR)			
	All female	Total sample 36.0 (11.4) (21–60)			
Ruwaard et al. (2013)	*N* = 105	Intervention 30.0 (10.0) (NR)	NR (Dutch community population)	NR	BN full syndrome/ sub‐threshold (no formal diagnoses)
	(35/35/35)	Bibliotherapy 31.0 (9.0) (NR)			
	99% female	Control 32.0 (11.0) (NR)			
		Total sample NR (NR) (NR)			
Sánchez‐Ortiz et al. (2011)	*N* = 76	Intervention 22.7 (3.1) (NR)	NR (59% British, 41% Other)	NR	BN/related EDNOS
	(38/38)	Control 25.0 (7.7) (NR)			
	98.7% female	Total sample 23.9 (5.9) (NR)			
Wagner et al. (2016)	*N* = 139	Intervention 34.9 (10.1) (18–59)	NR (German, Austrian, Swiss population)	NR	Threshold BED
	(69/70)	Control 35.3 (9.7) (19–61)			
	96.4% female	Total sample 35.1 (9.9) (18–61)			
**Relapse prevention studies**					
Fichter et al. (2012, 2013)	*N* = 258	Intervention 23.8 (6.5) (NR)	NR (German population)	NR	Threshold/ sub‐threshold AN (related EDNOS)
	(128/130)	Control 24.1 (5.6) (NR)			
	All female	Total sample NR (NR) (NR)			
Jacobi et al. (2017)	*N* = 253	Intervention 25.7 (7.2) (NR)	NR (German population)	NR	Threshold BN
	(126/127)	Control 26.3 (7.0) (NR)			
	All female	Total sample NR (NR) (NR)			

Abbreviations: AN, anorexia nervosa; BN, bulimia nervosa; BED, binge‐eating disorder; DSM, Diagnostic and Statistical Manual of Mental Disorders: DSM‐4 (American Psychiatric Association, 2000); DSM‐5 (American Psychiatric Association, 2013); DTC, delayed‐treatment control; EDNOS, eating disorders not otherwise specified; NR, not reported; SD, standard deviation; TAU, treatment‐as‐usual; WLC, waiting‐list control.

Studies were conducted in the UK, USA and mainland Europe. Although Beat (2015) reported that 25% of those with EDs are male, most participants in the included studies were female. The prevention studies included young college or university students recruited via higher education institutions, but in the treatment and relapse prevention studies the participants were slightly older clinical samples, except for one treatment study which used a student population (Sánchez‐Ortiz et al., 2011). The duration of prevention studies was 8–10 weeks, whereas treatment and relapse prevention studies were longer (3–9 months), highlighting that early intervention would benefit both patients and the healthcare system (Table 4).

Internet‐based CBT prevention programmes focused on reducing risk factors (e.g., fear of gaining weight) and improving protective factors (e.g., self‐esteem, coping mechanisms and quality of life). Eating disorder prevention studies that decrease the onset of EDs are sparse, most likely due to the difficulty in generating large sample sizes for sufficient power in such studies and lengthy follow‐up (Le et al., 2017). Moreover, clinical diagnostic interviews would be needed to confirm the development of a threshold ED, which would increase the costs of these studies and undermine the anonymity involved in ICBT programmes. The ICBT treatment programmes aimed to improve ED attitudes and behaviour as did the ICBT relapse prevention programmes, which sought to maintain the gains of hospital treatment and prevent relapse after discharge.

### Confounding variables

Comorbidities, such as depression, various forms of anxiety, stress, substance misuse and general psychopathology, were present in the samples, allowing some generalisability whilst introducing possible confounders (Carrard et al., 2011; Fichter et al., 2012; Ruwaard et al., 2013; Saekow et al., 2015; Sánchez‐Ortiz et al., 2011; Taylor et al., 2006, 2016; Wagner et al., 2016). Severe mental illnesses—major depression, psychosis and suicidal ideation—were excluded in all but three studies (Low et al., 2006; Winzelberg et al., 2000; Zabinski et al., 2001) so the results cannot be extrapolated to a broader population. Targeting baseline comorbidities with additional ICBT modules, preferably prior to starting an ICBT ED programme, may positively affect outcomes.

Co‐interventions such as stable dose of antidepressants was not prevented in three studies, which is aligned with routine clinical practice, but may possibly confound results (Carrard et al., 2011; Ruwaard et al., 2013; Sánchez‐Ortiz et al., 2011). Differential use of co‐interventions is of most concern. Antidepressants (or other psychoactive medications) are sometimes used alongside psychotherapy to treat EDs (Treasure et al., 2003, pp. 313–317), and their use during a study would raise concerns that improvements in participants may be partially or even entirely due to the drugs.

Compliance with the programmes, a possible confounder, was moderate to high across the prevention and treatment studies but was quite low in both relapse prevention studies which maybe indicates they were less acceptable to participants. Improving compliance could lead to better outcomes. It is suggested that factors such as baseline ED symptom severity; type of ED diagnosis; information technology (IT) difficulties; absence of face‐to‐face contact; and confidentiality issues may affect compliance and this needs to be considered.

Attrition was a concern in the included studies. Beintner et al. (2014) proposed that for self‐help interventions, treatment adherence is likely to be associated with better outcome. A good working alliance could reduce dropout rates from ICBT (Melville et al., 2010) but may take longer to form than in face‐to‐face therapy (Bengtsson et al., 2015). Melville et al. (2010) reported the average dropout rate for Internet‐based treatment for a wide range of psychological disorders to be 31% (with a range of 2%–83%). Despite being high, this is comparable to dropout rates of between 30% and 60%, for face‐to‐face psychotherapy (Garfield, 1994; Thormahlen et al., 2003; Wierzbicki & Pekarik, 1993). Monetary incentives (ranging from $25 to €80 for completing assessments) may have increased motivation in some participants but adding motivational interviewing modules could be more effective in improving adherence and ultimately the outcomes (Saekow et al., 2015). Aligned with Dölemeyer et al. (2013), there was no clear association between anonymity and dropout rates.

In most studies participants had a history of ED and/or past ED treatment which could be considered as possible confounders. A history of ED was not reported in just five studies (Low et al., 2006; Saekow et al., 2015; Taylor et al., 2006; Winzelberg et al., 2000; Zabinski et al., 2001). Similarly, past ED treatment was not reported in merely five studies (Fichter et al., 2012; Low et al., 2006; Saekow et al., 2015; Winzelberg et al., 2000; Zabinski et al., 2001). A negative past experience of an ED or its treatment may reduce expectations for successful treatment and adversely bias the results of studies (Newton & Ciliska, 2006).

Treatment credibility could influence uptake and study outcomes, as Internet treatments may be less credible than face‐to‐face treatments to some individuals (Andersson, 2009). A survey examining interest in behavioural and psychological interventions revealed that 92% of patients in primary care would opt for face‐to‐face treatment compared to 48% choosing Internet‐based treatment (Mohr et al., 2010). Similarly, Peynenburg et al. (2020) reported a greater preference for face‐to‐face CBT rather than ICBT (44.6% vs. 23.5%) for the treatment of depression and anxiety in students aged 18–61 years. Other studies proposed conflicting results, for example, Cavanagh et al. (2006) suggested that 20%–33% of patients declined ICBT programmes in comparison to 25%–50% of patients forgoing face‐to‐face psychotherapy after initial assessments; this implies that ICBT programmes could be slightly more appealing to patients than traditional treatments. Furthermore, patients and general practitioners (GPs) seem to find ICBT more acceptable than do therapists (Waller & Gilbody, 2008).

### Meta‐analyses

Statistically significant summary and individual effect sizes (*p* ≤ .05), extent of heterogeneity (*I* ^2^) and GRADE ratings for the included studies are presented in Table 5.

**TABLE 5:** Meta‐analyses: statistically significant effect sizes.

Outcome	Effect size SMD = g (95% CI) *I* ^2^%	GRADE rating
**Prevention studies: At‐risk patients**		
EDE‐Q		
Eating disorder		
Cognitive symptoms		
Restraint	**−0.41 (−0.80, −0.02)** 75	Very low
Weight concern	**−0.31 (−0.57, −0.06)** 50	Very low
Global score	**−0.46 (−0.82, −0.09)** 79	Very low
EDI/EDI (2)		
Drive for thinness	**−0.45 (−0.61, −0.28)** 26	Low
WCS	**−0.47 (−0.82, −0.11)** 80	Very low
**Treatment studies: (Bulimia nervosa [BN] and binge‐eating disorder [BED] patients)**		
EDE‐Q		
Eating disorder		
Cognitive symptoms		
Eating concern	**−0.99 (−1.34, −0.63)** NA	Very low
Weight concern	**−1.11 (−1.47, −0.75)** NA	Very low
Shape concern	**−0.65 (−1.29, −0.01)** 80	Very low
Global score	**−0.70 (−1.22, −0.17)** 77	Very low
EDE‐Q		
Behavioural items		
Binge rate	**−0.66 (−1.11, −0.22)** 69	Very low
EDI/EDI (2)		
Bulimia	**−0.85 (−1.33, −0.37)** NA	Very low
Interoceptive awareness	**−0.51 (−0.97, −0.05)** NA	Very low
BDI/CES‐D	**−0.47 (−0.74, −0.20)** 0	Very low
SCL‐90R	**−0.30 (−0.57, −0.03)** 0	Very low
**Relapse prevention study: (Anorexia nervosa [AN] and related eating disorder not otherwise specified [EDNOS] patients)**		
EDI/EDI (2)		
Maturity fears	**−0.43 (−0.70, −0.16)** NA	Low
Social insecurity	**−0.29 (−0.56, −0.03)** NA	Low

*Note*: The bold values indicates the effect sizes (SMD with 95% CI).

Abbreviations: 95% CI, 95% confidence interval; BDI/BDI‐2, Beck depression inventory (Beck et al., 1988); CES‐D, Centre for Epidemiological Studies Depression scale (Orme et al., 1986); EDE‐Q, eating disorder examination questionnaire (Fairburn & Beglin, 1994); EDI/EDI‐2, eating disorder inventory (Garner & Olmsted, 1984); GRADE, grades of recommendation, assessment, development and evaluation ratings; *I* ^2^, extent of heterogeneity (inconsistency between studies); NA, not applicable; SMD, standardised mean difference, in this case, Hedges' (adjusted) *g*; SCL‐90R (GSI), Symptom checklist 90R, general symptom index score–general psychopathology/anxiety (Derogatis, 1975); WCS, weight concern scale (Killen et al., 1994).

Negative effect estimates show that the intervention is superior to control and vice versa. A null value of zero indicates equivalence for the two groups. The 95% confidence interval (CI) is the range within which the true value can be found 95% of the time. If the CI includes the null value, the effect estimate is not significant (*p* > .05). When the CI excludes the null value, the effect estimate is statistically significant (*p* ≤ .05); it is unlikely to be due to chance and the null hypothesis of no difference between the groups can be rejected (Higgins & Green, 2008, pp. 369–372).

Most of the effect sizes for all three intervention subgroups (prevention, treatment and relapse prevention) were imprecise as evident from the very wide confidence intervals, and non‐significant beneficial effect sizes may be due to insufficient power. Improvements in the control group may be explained by life events, regression towards the mean, maturation leading to a more balanced perspective of shape, weight and body dissatisfaction, or due to ED campaigns on campus, online or in the community.

Sensitivity analyses demonstrated that *indicated* prevention studies had larger effect sizes than *universal* prevention studies, suggesting that ICBT is more effective for participants at higher risk—which is in parallel with Watson et al. (2016). ‘Intensive’ studies with increased therapist contact (i.e., Ruwaard et al., 2013; Wagner et al., 2016) showed more favourable results. Additionally, BED may be easier to treat than BN as the effect sizes for the former were larger; this is supported by Ljotsson et al. (2007). These are exploratory analyses and need to be interpreted with caution.

There was a pattern of very high heterogeneity (*I* ^2^) across the meta‐analyses, thus lowering confidence in the combined results. Heterogeneity could be improved via the use of similar ICBT programmes and participants especially regarding risk of ED or type of ED. Analyses undertaken reinforced that publication bias was not of great concern in this study and the search strategy was exhaustive, but searching grey literature would have increased confidence in the results.

These results are limited by the small number of included studies. The GRADE ratings for the outcomes were mostly very low and decrease the credibility of the results. Despite the drawbacks, the results of this systematic review are likely to be the best in the field so far as the data is derived exclusively from RCT studies—the best evidence base available.

## DISCUSSION

### Is ICBT efficacious?

This systematic review evaluated the efficacy of ICBT interventions for all types of EDs in adults. Findings typically showed medium significant beneficial effect sizes for prevention studies ranging from (−0.31 [95% CI: −0.57, −0.06] to −0.47 [95% CI: −0.82, −0.11]) and generally large effect sizes for the treatment studies ranging from (−0.30 [95% CI: −0.57, −0.03] to −1.11 [95% CI: −1.47, −0.75]). Relapse prevention yielded mainly small non‐significant beneficial effect sizes with significant effect sizes of (−0.29 [95% CI: −0.56, −0.03] and −0.43 [95% CI: −0.70, −0.16]).

In the treatment studies, 35% of effect estimates exceeded the minimally important difference (MID) SMD = 0.5 (Norman et al., 2003); thus, these results were clinically significant and were evident for the *Eating Disorder Examination‐Questionnaire*'*s* (EDE‐Q) cognitive symptoms (eating, weight and shape concerns, and global score); the EDE‐Q's behavioural item (binge rate); the *Eating Disorder Inventory*'*s* (EDI) bulimia (binge‐eating and purging behaviour) and interoceptive awareness (sensations of hunger and satiety). None of the effect estimates for either the prevention or the relapse prevention studies reached clinical significance. This could be due to the greater scope for improvement in the participants of the treatment studies.

The moderate favourable influence on depression and a small positive impact on general psychopathology/anxiety for the treatment programmes, which were not transdiagnostic, are important as EDs are frequently comorbid with depression or anxiety (Beat, 2015).

Internet‐based CBT influenced cognitive symptoms (e.g., eating and weight concerns) more than it affected behavioural symptoms (e.g., binge or purge rates). By contrasting the effect sizes of this review with those provided by Linardon et al. (2017), it is suggested that ICBT may be more efficacious than face‐to‐face CBT for treating cognitive symptoms. This may be because participants have more time to absorb the cognitive content in ICBT than in face‐to‐face CBT. Improvements in both behavioural and cognitive symptoms are imperative as residual cognitive symptoms may lead to relapse (Linardon et al., 2017). If the impact on cognitive symptoms leads to lower relapse rates, then the long‐term effectiveness of ICBT would be significantly more effective than other treatments. While direct comparisons between ICBT and face‐to‐face CBT are needed, these findings are very encouraging.

For the ICBT ED prevention programmes, the evidence base that supports ICBT's efficacy, although it varies in quality and is moderate in size, still provides reasonable confidence in these programmes. This is in spite of the use of programme completers' data in the meta‐analyses, which may present biased over‐estimates. The very large effect sizes found for the treatment programmes is countered by some quality concerns and a limited evidence base. Even though the two relapse prevention programmes are of high quality, the evidence base for these programmes is very small. It is difficult to reach conclusions about the treatment and relapse prevention programmes although the more cautious intention‐to‐treat (ITT) results were used in the meta‐analyses. Many more high‐quality ICBT ED RCTs are needed to enable precise meta‐analyses and robust conclusions.

Internet‐based CBT for prevention is a difficult issue for dissemination as most individuals remain at some risk after prevention trials, and resources are simply not available for people to then go to a higher level of care, like face‐to‐face. Further, Taylor et al. (2021) provide a broad summary of the evidence supporting the effectiveness of Internet interventions and describe lower effect sizes for EDs and substance abuse compared to depression and anxiety. Nevertheless, this review provides preliminary evidence that ICBT interventions could be offered to adult community and student populations in Western countries in an adaptive stepped care approach and is likely to be cost‐effective compared to face‐to‐face CBT. Such an adaptive stepped care approach would be more flexible and tailored to individual needs. Patients could then be monitored closely and receive more intensive support earlier or when indicated. The programme ESSPRIT (Bauer et al., 2009) and its Dutch translation Featback (www.featback.nl) are examples of an adaptive stepped care approach.

Comparison with previous reviews shows some support for the results of the current review. A few meta‐analyses have investigated prevention studies and some have looked at treatment and relapse prevention studies but these studies did not focus entirely on ICBT. Beintner et al. (2012) reported small to medium improvements in ED psychopathology (cognitive symptoms) in a review of prevention studies. Harrer et al. (2020) also reported small to medium effect sizes under the prevention category. Aardoom et al. (2013) reviewed treatment studies and found little impact on purging behaviour but improvements in quality of life, binge‐eating and especially ED psychopathology. Similarly, Dölemeyer et al. (2013) in their review of treatment studies described medium to large effect sizes for ED symptoms and improvements in abstinence, quality of life, depression and anxiety. Both Bauer and Moessner (2013) and Schlegl et al. (2015) examined the efficacy of prevention, treatment and relapse prevention programmes, with the former purporting ‘promising’ results and the latter detailing small to large and large effect sizes for behavioural and cognitive symptoms, respectively. Loucas et al. (2014) found small improvements in ED psychopathology for prevention, but treatment and relapse prevention results were inconclusive. The most recent review is by Linardon et al. (2020) and has reported positive results for prevention and treatment studies.

After this review was completed, a cluster randomised RCT of ICBT versus usual care for women with binge‐purge EDs was found which showed significant reduction in eating disorder psychopathology in the intervention group compared to control at postintervention and at follow‐up (Fitzsimmons‐Craft et al., 2020). Another study, which has not been published, also addresses a core issue of this review (Jacobi et al., 2023): RCT analysing indicated, Internet‐based prevention for women with anorexia nervosa (AN) symptoms.

At the time of initiation, this review updated the Loucas et al. (2014) study and included five new RCT studies. Subsequently, Linardon et al. (2020) published their study but did not include relapse prevention studies and looked at e‐therapies more broadly rather than focusing on ICBT. In agreement with this systematic review, these two latest and most comprehensive systematic reviews on the efficacy of e‐mental health ED programmes have taken an optimistic perspective, while clarifying the need for further research. Interestingly, larger effect sizes were found in the current review compared to these two reviews. This could be due to focusing exclusively on RCTs and Internet‐delivered CBT and indicating that at this time ICBT may be more effective for treating EDs than other forms of Internet psychotherapies and delivery via downloadable software, CD‐ROMs and mobile applications. The strengths of this review are arguably the inclusion of the best evidence base (just RCTs), specifically ICBT programmes, all types of EDs and solely adult participants. Notably, these characteristics are not addressed together in previous reviews and as such this review likely provides clearer estimates of the efficacy of ICBT for EDs in adults.

### Limitations

The main drawback of this systematic review is that only an independent researcher conducted the review processes; however, this was under supervision by an academic lecturer. Other deficits include that long‐term effects could not be explored statistically as the follow‐up periods were disparate in the studies, and grey literature was not searched but it is contended that the search was exhaustive. Although confidence in the results of this review is attenuated by the small number of included studies, this is the best evidence base to date.

### Future research

More high‐quality RCTs are needed, particularly those that compare ICBT to active comparisons and effectiveness studies in order to strengthen the case for dissemination and implementation of ICBT for EDs. RCTs should accord with the CONSORT statement (Schulz et al., 2010) and researchers should follow the guidance for conducting and reporting ICBT research (Proudfoot et al., 2011). Such research will facilitate more precise meta‐analyses to reach unequivocal conclusions. Replication of the large effect sizes demonstrated for the treatment programmes would support the necessity of developing these programmes further. Including non‐English language studies could have retrieved RCTs from populations that do not primarily speak English (e.g., individuals living in Asia, Africa, South America). However, at this time the generalisability of ICBT programmes included in this systematic review is limited predominantly to young Caucasian women in Western countries. Further research is essential to confirm whether the findings can be transferred to other ethnicities, countries beyond the UK, USA and mainland Europe, and different healthcare systems.

In order to increase resources, decrease waiting times and costs, it is necessary to understand which patient groups would benefit most from ICBT, and those who require more intensive support. Research is required to clarify how much time patients need to allocate to the ICBT programme to maximise outcomes, the optimal intensity of therapist support, and whether the support provided by non‐clinicians can be equally effective.

Patient safety needs to be explicitly investigated and systems should be put in place to support patients experiencing a crisis, before ICBT can be considered as a viable option. Online or mobile app support networks could be incorporated into ICBT programmes to mitigate the distress arising from delayed response to crises. The development of strategies to monitor and alert to non‐responders and deteriorating conditions are required (i.e., integrating automated regular monitoring of symptoms and feedback into ICBT programmes).

Finally, the use of digital technology (computers, tablets, mobile phones) is prevalent worldwide and has the potential to extend the reach of mental health interventions even in low‐ and middle‐income countries (Lehtimaki et al., 2021). As Taylor et al. (2020) outlined, the COVID‐19 pandemic has led to a shift in using telehealth instead of face‐to‐face consultations and highlighted the need for digital mental health interventions to become part of routine care. Barriers to digital services (e.g., training of therapists, licensing regulations, patient safety concerns, ensuring the privacy of patients, reimbursement for digital therapies, and further research into the gaps in knowledge about digital therapies) need to be addressed, and it is necessary to exploit new technologies, including virtual or augmented reality, to optimise the content and delivery of ICBT ED programmes with the goal of providing timely, flexible, personalised, interactive, cost‐effective and evidence‐based interventions.

## CONCLUSIONS

This systematic review reinforces the vital need to provide evidence‐based Internet interventions at times when face‐to‐face treatment is not an option as was the case during the COVID‐19 pandemic. Minimally guided self‐help ICBT programmes, without face‐to‐face therapy, appear to be efficacious and have shown improvements in ED risk factors, onset, symptoms and relapse in adults. However, a demand for further high‐quality RCT research on ICBT for EDs was identified and future research needs to ascertain both short‐ and long‐term effectiveness, cost‐effectiveness, patient safety and acceptability of ICBT to improve these programmes and maximise benefits to patients.

## FUNDING INFORMATION

No financial support was provided for this systematic review.

## CONFLICT OF INTEREST STATEMENT

The authors declare no conflicts of interest.

## ETHICS STATEMENT

This work was secondary research (a systematic review) and did not involve the use of human subjects; thus, there were no ethical considerations.

## Data Availability

The data that support the findings of this study are available from the corresponding author upon reasonable request.
